# Physiotherapy management of patients undergoing thoracotomy procedure: A survey of current practice in Gauteng

**DOI:** 10.4102/sajp.v73i1.344

**Published:** 2017-08-28

**Authors:** Liezel Schwellnus, Ronel Roos, Vaneshveri Naidoo

**Affiliations:** 1 Physiotherapy Department, Faculty of Health Sciences, University of the Witwatersrand, South Africa

## Abstract

**Background:**

Physiotherapy is included in the management of patients undergoing thoracic surgery. The aim of this study was to describe physiotherapy practice in the management of patients who undergo an open thoracotomy.

**Methods:**

A cross-sectional study using convenience sampling was undertaken. An electronic self-administered questionnaire was distributed via SurveyMonkey to 1389 physiotherapists registered with the South African Society of Physiotherapy in Gauteng. The data collection period was August and September 2014 and data were analysed descriptively.

**Results:**

A total of 323 physiotherapists (23.3%) responded to the survey and 141 (10.2%) indicated that they treated patients with open thoracotomies. Preoperative treatment was done by 65 (41.6%) and consisted of teaching supported coughing (92.3%; *n* = 60), sustained maximal inspiration (70.8%; *n* = 46) and the active cycle of breathing technique (69.2%; *n* = 45). One hundred and sixteen (82.3%) respondents treated patients during their hospital stay. Deep breathing exercises (97.6%; *n* = 83), coughing (95.3%; *n* = 81), early mobilisation (95.3%; *n* = 81), upper limb mobility exercises (91.8%; *n* = 78), chest wall vibrations (88.2%; *n* = 75) and trunk mobility exercises (85.9%; *n* = 73) were done frequently. Pain management modalities were less common, for example transcutaneous electrical nerve stimulation (12.9%; *n* = 11). Post hospital physiotherapy management was uncommon (32.6%; *n* = 46).

**Conclusion:**

Physiotherapy related to early mobilisation in hospital is in line with evidence-based practice, but further education is needed regarding the need for physiotherapy post hospital discharge and pain management.

## Introduction

Open thoracotomy is a surgical incision in the chest wall to open and gain access to the thoracic cavity (Lazopoulos et al. 2015). It is used for the management of a wide range of conditions such as pulmonary malignancies, pulmonary infections, chest trauma (e.g. plating of flail rib segments), bullous disease, intrapleural disorders, etc. (El-Ansary, Reeve & Denehy 2016). Patients in South Africa who undergo open thoracotomies often include individuals with severe lung diseases or chest trauma (Calligaro et al. 2014; Clarke et al. 2011). The standard surgical approach is the posterolateral incision which provides the best visibility of structures but results in significant post-operative pain compared with other incisions such as the anterolateral and muscle sparing techniques (Li et al. 2014; Yamaqhuci, Hashimoto & Tamaki 2006). This approach is known to result in more shoulder dysfunction and post-operative pain in patients (El-Ansary et al. 2016). Reduced physical activity on Day 1 post-open thoracotomy is noted to be because of post-operative pain and reduced physical activity at Day 6 is linked with increased incidence of post-operative pulmonary complications (PPCs) (Agostini et al. 2014). Effective pain management following open thoracotomy is thus necessary to ensure patient comfort, lessen PPCs and enhance physical recovery (De Cosmo et al. 2009).

A dearth of literature exists that describes physiotherapy management of such patients. Two studies were sourced: one study was conducted in Australia and New Zealand by Reeve et al. (2007) and another by Agostini et al. (2013) in the United Kingdom (UK). The respective authors noted that physiotherapy services are most often offered to patients when in hospital but less so following hospital discharge. This is cause for concern as functional recovery following open thoracotomy is known to be slower compared with when a patient undergoes video-assisted thoracoscopic surgery (VATS) (Shi et al. 2016). Shi et al. (2016) noted that patients following open thoracotomy only return to preoperative walking ability after 43 days compared with 18 days in individuals who had undergone a VATS procedure. Symptoms that influence recovery include fatigue, pain, shortness of breath, disturbed sleep and drowsiness (Fagundes et al. 2015). Exercise training as part of the rehabilitation process for patients following lung resection for non-small cell lung cancer is beneficial in improving patients’ walking distance and exercise capacity (Cavalheri et al. 2014). Continuation of physiotherapy management following hospital discharge could influence some of these remaining symptoms and thus impact on individuals’ functional recovery.

During hospital stay, physiotherapists focus on prevention and management of PPCs and improving thoracic and shoulder mobility of patients (Agostini et al. 2013; Arbane et al. 2011; Reeve et al. 2007). Treatment modalities to address PPCs include breathing exercises (active cycle of breathing technique [ACBT], deep breathing exercises [DBE], incentive spirometry, sustained maximal inspiration, intermittent positive pressure breathing and positive expiratory pressure devices), cough manoeuvres and forced expiratory technique (Agostini et al. 2013; Reeve et al. 2007). In addition, early progressive functional activities such as sitting out of bed, marching on the spot, walking, stair climbing and cycling are included as treatment options to address PPCs and improve physical function (Agostini et al. 2013; Reeve et al. 2007). Maintaining shoulder and trunk mobility is carried out by performing shoulder and trunk range of motion exercises (Agostini et al. 2013; Reeve et al. 2007).

No published literature could be sourced that describes how physiotherapists working in the South African context are managing such patients. The aim of this study was thus to determine the current physiotherapy practice with regard to the management of patients who undergo an open thoracotomy procedure.

## Methodology

### Study design and sample selection

A sample of convenience, specifically all physiotherapists registered with the South African Society of Physiotherapy (SASP) in Gauteng (*n* = 1389), was sent an electronic self-administered questionnaire using SurveyMonkey.

### Procedure

The original questionnaire used by Reeve et al. (2007) was adapted following permission received from the first author to use the questionnaire in this study. The questionnaire was peer reviewed and piloted to evaluate the estimated time taken to answer the questionnaire, review if questions were understandable and relevant in a South African context and identify if there were any problems with the SurveyMonkey programme when completing the questionnaire. Invitations to participate in the study were sent out to physiotherapists electronically via SASP communication and the link to the SurveyMonkey questionnaire was included in these communications. Physiotherapists were invited to participate in the study if they managed patients who had or were having open thoracotomies. The data collection period was two months during 2014 (August and September). Data analysis was performed using IBM SPSS version 22 (IBM Corp 2013). Categorical parameters were summarised using frequencies and percentages. Means and standard deviations were determined for the demographic variables of age and years qualified.

### Ethical considerations

Ethical clearance was obtained from the University of the Witwatersrand Human Research Ethics Committee (certificate number M140414) and permission was granted by the South African Society of Physiotherapy for distribution of the survey to its members.

## Results

Of the 1389 circulated invitations, 323 physiotherapists (23.3%) responded to the survey. Of the 323 responses received, 43.7% (*n* = 141) indicated that they treat patients who received open thoracic surgery, and 176 were excluded during the data analysis of the study as these respondents indicated that they do not treat such patients. Six additional respondents’ data were excluded during analysis because of not indicating if they treated such patients. Thus of the 1389 study invitations circulated, only 141 (10.2%) respondents indicated that they treat the population of interest.

### Demographic profile of physiotherapist

The majority of respondents were female (95.7%; *n* = 135), had a bachelor’s degree in physiotherapy (80.1%; *n* = 113), were employed in the private sector (87.2%; *n* = 123) and worked in an inpatient and outpatient setting (59.0%; *n* = 83) (Table 1).

**TABLE 1:** Demographic profile of physiotherapists treating patients with a thoracotomy incision (*n* = 141).

Variable	Frequency (%)	Mean (±SD)
Age, years	-	35.9 (11.1)
Number of years qualified	-	13.4 (11.1)
Gender		-
• Male	6 (4.3)	
• Female	135 (95.7)	
Highest qualifications		-
• Diploma in Physiotherapy	9 (6.4)	
• Bachelor’s in Physiotherapy	113 (80.1)	
• Master’s in Physiotherapy	17 (12.1)	
• Doctorate in Physiotherapy	2 (1.4)	
Attendance of continuous development activities (CPD)		-
• Yes	139 (98.6)	
• No	2 (1.4)	
Categories of CPD attended during the previous year		-
• Cardiorespiratory	51 (36.7)	
• Orthopaedic manipulative therapy	76 (54.7)	
• Sport	47 (33.8)	
• Pain	46 (33.1)	
• Other	41 (29.5)	
Employment status		-
• Academic	3 (2.1)	
• Private sector	123 (87.2)	
• Public sector	6 (4.3)	
• Private and public sector	9 (6.4)	
Physical workplace setting		-
• Outpatients and inpatients	83 (59.0)	
• Inpatients	27 (19.0)	
• Outpatients	24 (17.0)	
• Academic and clinical	7 (5.0)	

*Source*: Authors’ own work

### Physiotherapy provision of care

Of the 141 respondents, 95 (67.4%) treated patients during their hospital stay, 25 (17.4%) provided this service to patients following discharge from hospital and 21 (14.9%) respondents noted treating patients during and after their hospital stay. Reasons for open thoracotomies at their respective institutions were varied: elective pleural surgery (55.3%; *n* = 78), elective pulmonary resection (53.2%; *n* = 75), open thoracotomy because of trauma-related injury (31.2%; *n* = 44), elective oesophageal surgery (22.7%; *n* = 32), elective lung volume reduction surgery (18.4%; *n* = 26), non-traumatic chest wall reconstructive surgery (15.6%; *n* = 22) and other reasons, for example valve replacement surgery (10.6%; *n* = 15). If elective open thoracotomy was performed, the majority of respondents (22.3%; *n* = 72) reported that length of hospital stay was 4–7 days. If open thoracotomy was performed because of trauma-related injury most respondents (7.7%; *n* = 25) noted that hospital length of stay for these patients was more than 10 days.

### Preoperative physiotherapy management

Of the 141 respondents, 65 (46.1%) did preoperative patient management. The procedures most often used to evaluate patients preoperatively by these therapists were review of X-rays (96.9%; *n* = 63), auscultation (92.3%; *n* = 60), review of clinical notes (89.2%; *n* = 58) and screening of the patient’s functional ability (81.5%; *n* = 53). Endurance capacity testing, for example 6-min walk test, was the assessment skill least likely to be performed (15.4%; *n* = 10). Preoperative treatment mainly consisted of teaching supported coughing and huffing (92.3%; *n* = 60), teaching sustained maximal inspiration (70.8%; *n* = 46), ACBT (69.2%; *n* = 45) and incentive spirometry (60.0%; *n* = 39). Inspiratory muscle training (IMT) was done less often (40.0%; *n* = 26).

### Post-operative physiotherapy management in hospital

Of the 141 respondents, 116 (82.3%) treated patients during hospital stay. Of the 116 respondents, 85 individuals specified their interventions used. The six treatment modalities used by most of these respondents were: DBE (97.6%; *n* = 83), coughing (95.3%; *n* = 81), early mobilisation (95.3%; *n* = 81), upper limb mobility exercises (91.8%; *n* = 78), chest wall vibrations (88.2%; *n* = 75) and trunk mobility exercises (85.9%; *n* = 73). When detailing ‘early mobilisation’ all of these respondents indicated that they assisted patients to sit in a chair and with walking (100%; *n* = 85) and 73 (85.9%) included stair climbing as well. Transcutaneous electrical nerve stimulation (TENS) was utilised infrequently (12.9%; *n* = 11). Prior to hospital discharge 31.8% (*n* = 27) of respondents provided patients with an education discharge booklet and 95.3% (*n* = 81) of respondents provided home advice verbally. The majority of these respondents (64.7%; *n* = 55) indicated that they sometimes referred patients for treatment following hospital discharge and 12 (14.1%) indicated that they always did so. Reasons for referral included respiratory difficulties (58.8%; *n* = 50), decreased functional ability (54.1%; *n* = 46), decreased shoulder and trunk mobility (41.2%; *n* = 35), patients still experiencing pain (41.2%; *n* = 35) and reduced cardiovascular endurance (38.8%; *n* = 33).

### Post-operative physiotherapy management after discharge from hospital

Of the 141 respondents, 46 (32.6%) treated patients after hospital discharge. The major referral source was self-referral by patients (32.6%; *n* = 15). Reasons for physiotherapy management during the first six weeks after hospital discharge were: reduced cardiovascular endurance (34.8%; *n* = 16), decreased functional ability (32.6%; *n* = 15), patients still complaining of pain (26.1%; *n* = 12), decreased trunk and shoulder mobility (21.7%; *n* = 10), respiratory difficulties (13.0%; *n* = 6) and other reasons, for example muscle spasm and neck pain (10.9%; *n* = 5). Reasons for physiotherapy management more than six weeks following hospital discharge were as follows: patients still complaining of pain (41.3%; *n* = 19), decreased functional ability (41.3%; *n* = 19), decreased trunk and shoulder mobility (36.9%; *n* = 17), respiratory difficulties (19.6%; *n* = 9), reduced cardiovascular endurance (13.0%; *n* = 6) and other reasons, for example neck and thoracic spasm (2.1%; *n* = 1). The six treatment techniques most often utilised after hospital discharge were the following: upper limb mobility exercises (65.2%; *n* = 30), soft tissue treatment techniques to increase mobility (63.0%; *n* = 29), general exercises (63.0%; *n* = 29), soft tissue treatment techniques to reduce pain (60.9%; *n* = 28), trunk mobility exercises (58.7%; *n* = 27) and progressive walking programmes (56.5%; *n* = 26). Electrotherapy modalities to decrease pain were used infrequently (30.3%; *n* = 14).

## Discussion

This study provides information on physiotherapy management of patients who undergo an open thoracotomy procedure in Gauteng. Results indicated that physiotherapy management was carried out by the majority of respondents (82.3%) during hospital stay and less so preoperatively (46.1%) or after hospital discharge (32.6%). This trend is in line with findings reported by Reeve et al. (2007) and Agostini et al. (2013).

An encouraging finding of the study was that early mobilisation activities (sitting in a chair and walking) were utilised as treatment modality by all respondents who treated patients during hospital stay. A reduction of 131.6 (±101.8) m in patients’ 6-min walk test distance can occur from the day before surgery to five days following an open thoracotomy (Arbane et al. 2011). Walking less post-operatively is cause for concern considering the link with PPCs. Agostini et al. (2014) noted patients who were less active (took less than 500 steps during Day 2–8 after surgery) had a higher incidence of developing PPCs compared with more active patients (20% vs 4%; *p* = 0.034). Thus, including early mobilisation activities as was done in this study may be beneficial in preventing PPCs and reducing the proposed decline in walking distance of patients post-operatively.

In addition to early mobilisation activities, respondents included DBE, cough manoeuvres, upper limb exercises and trunk mobility as part of post-operative physiotherapy treatment. A recent study conducted by Rodriguez-Larrad et al. (2016) found that including deep breathing, cough manoeuvres, upper limb and trunk exercises as a physiotherapy treatment regime in patients following open thoracotomy lessened the incidence of PPCs in an intervention group (6.6%) when compared with a control group (20.6%). Length of hospital stay in the authors’ study was also less in the intervention group (12 [±6] days) compared with the control group (14 [±7] days). Interestingly, the study found that the said physiotherapy regime significantly reduced the development of PPCs (β = -1.650; *p* = 0.007) but patient-controlled epidural analgesia had no influence on whether patients developed PPCs (β = 0.939; *p* = 0.153). It should be noted that early mobilisation was part of standard care at the specific hospital in the authors’ study and nursing staff assisted patients with mobilisation.

In contrast, Reeve et al. (2010) implemented a similar physiotherapy regime (deep breathing, cough manoeuvres, upper limb and trunk exercises and early mobilisation activities) but found the intervention had no effect on patients developing PPCs not did it influence their length of stay in hospital. The authors did note that their study population included individuals with good preoperative lung function and this might have influenced their findings. The authors highlighted that the above treatment programme reduced participants’ intensity of shoulder pain and the total score on the Shoulder Pain and Disability Index at discharge from hospital. In addition when study participants continued with the exercise programme after hospital discharge it improved their quality of life. This finding of the study is of value considering that shoulder dysfunction often develops in patients following an open thoracotomy because of pain (El-Ansary et al. 2016; Li et al. 2014; Yamaqhuci et al. 2006). Thus, implementing a post-operative physiotherapy treatment regime including the said modalities listed by Reeve et al. (2010) and Rodriquez-Larrad et al. (2016), as was done here, may influence patients’ length of hospital stay, the likelihood of patients developing PPCs and could reduce patients’ pain levels, improve their shoulder function and general quality of life.

A number of respondents (*n* = 44) managed patients who required open thoracotomy because of trauma-related injury. Patients who sustain blunt chest trauma often require surgical corrections of rib fractures via an open thoracotomy as a means of stabilising the chest wall (El-Ansary et al. 2016; Unsworth, Curtis & Asha 2015). This procedure is reported to be cost saving as it reduces length of stay of patients in intensive care and reduces the days spent on mechanical ventilation (Unsworth et al. 2015). A multidisciplinary team approach is suggested as the optimal way to manage such patients and treatment should focus on early mobilisation, DBE and aggressive pain management (Unsworth et al. 2015). The post-operative treatment (DBE and early mobilisation) provided by respondents are thus in line with suggested treatment principles recommended by Unsworth et al. (2015).

Preoperative physiotherapy treatment focused mainly on educating patients on supported cough, huff and breathing exercises. Published support for preoperative physiotherapy is limited. Reid et al. (2010) assessed the effect of patient education consisting of supported cough and huff, DBE, ankle pumps and shoulder range of motion exercises on the development of PPCs in patients following open thoracotomy. The authors found no effect on PPCs when including patient education as a preoperative modality. Inspiratory muscle training was utilised infrequently as a preoperative treatment here. A recent meta-analysis by Mans et al. (2014) concluded that preoperative IMT was effective in reducing PPCs leading to improved lung function early following surgery. Reeve et al. (2007) reported minimal usage of preoperative IMT with only one respondent using this intervention routinely in their study. Possible reasons for less utilisation of this technique in this study could be effort, time and cost associated with implementation, as well as the possible lack of knowledge of respondents regarding the effectiveness of IMT in reducing PPCs following surgery. In addition, trauma-related injury was one reason why open thoracotomies were performed at respondents’ hospitals. These patients may have been too unstable preoperatively for physiotherapy intervention compared to elective surgeries performed resulting in less utility of IMT.

Of the respondents, 32% provided a service to patients following discharge from hospital. Pain was reported as the third most prominent symptom in the first six weeks following discharge from hospital and the most important symptom for patients seeking physiotherapy treatment after six weeks after hospital discharge. Post thoracotomy pain syndrome (PTPS) is a common complication following thoracic surgery (Hopkins et al. 2015).

PTPS is defined as ‘pain that recurs or persists along a thoracotomy scar at least two months following the surgical procedure to the chest wall’ (International Association for the Study of Pain, Subcommittee of Taxonomy 1986). The incidence of PTPS has been reported in 25%–60% of patients (Della Corte et al. 2012; Gotoda et al. 2001; Humble, Dalton & Li 2015; Wildgaard et al. 2009). This high incidence of PTPS is indicative of early, effective post thoracotomy pain management required. The intensity of acute pain is a risk factor associated with the development of PTPS (Amprachim et al. 2013; Gotoda et al. 2001; Joshi et al. 2008; Wildgaard et al. 2009). Direct and indirect damage to the intercostal nerves appears to be the primary cause of pain, largely neuropathic pain, leading to central sensitisation and development of PTPS (Amprachim et al. 2013; Della Corte et al. 2012; Hopkins & Rosenweig 2012; Khelemskey & Noto 2012).

The persistence of pain (41.3%) in patients longer than six weeks following hospital discharge could be attributed to open thoracotomy as a result of trauma (31.2%) and scant pain management. Only 10 respondents utilised TENS during hospital stay as a means of treating pain. TENS is beneficial in lessening post-operative pain following thoracotomy and reported to improve the tolerance for physiotherapy treatment during hospital stay (Freynet & Falcoz 2010).

Treatment of respiratory complications was prioritised immediately post-operatively. Pain management came to the fore when patients sought physiotherapy treatment longer than six weeks after hospital discharge. The value of early pain management associated with decreased respiratory complications and paucity of chronic pain is emphasised by the following authors: Amprachim et al. 2013; Humble et al. 2015; Joshi et al. 2008; Pennefather & McKevith 2011; Wildgaard 2009. Function and quality of life, fundamental aspects of well-being, are adversely affected by acute and chronic pain (Della Corte et al. 2012; Hopkins et al. 2015; Pennefather & McKevith 2011). The aforesaid is demonstrated in this study, with decreased functional ability evident six weeks post hospital discharge and beyond (32.6% and 41.3%, respectively). Pain is a significant factor influencing respiratory function and well-being and therefore effective early pain management strategies must be incorporated into the rehabilitation of patients following thoracotomy.

## Limitations

Information gained from this study is limited to physiotherapists registered with the SASP in Gauteng province. Because of the poor response rate, the study only gives a glimpse of current practice in South Africa. The majority of respondents (93.6%; *n* = 132) indicated that they work in the private health care sector and therefore information regarding physiotherapy management of patients following open thoracotomy in the public sector is lacking. In hindsight, more information regarding the reasons why not all patients are seen post-operatively, as well as the possible assessment procedures performed, would have given valuable insight into the post-operative management of patients while still in hospital. A clearer South African picture might have emerged if respondents had been asked to differentiate between techniques used after elective thoracotomy versus emergency thoracotomy. More in-depth questioning on why certain techniques are preferred might have revealed that more physiotherapists than estimated in this study could indeed be involved in early interventions to prevent PTPS.

## Conclusions

Indications for physiotherapy in this patient population are often to prevent or decrease the incidence of PPCs and to manage physical recovery of patients if complications are present. The modalities used most commonly by respondents were respiratory techniques as well as early mobilisation. Exercise interventions were used to manage shoulder and thoracic cage mobility. A limited number of modalities focused on treating pain, with this part of patient management seemingly left to be controlled by analgesics. Physiotherapy after hospital discharge was infrequent and more active involvement of physiotherapists during this phase of rehabilitation should be encouraged considering the problems that were present in patients at hospital discharge. A more active role in the management of patients’ pain should also be advocated, as a decrease in pain leads to better respiratory mechanics resulting in a reduction of not only PPCs but also of the incidence of PTPS (Joshi et al. 2014). Studies evaluating the effect of physiotherapy management implemented in this population after hospital discharge would be of added value. In addition, studies evaluating the effect of physiotherapy management of patients following plating of flail segments would be of value considering the scarcity of published literature available.
